# Supplementary material for: Microbiological Aspects and Enzymatic Characterization of Curvularia kusanoi L7: Ascomycete with Great Biomass Degradation Potentialities
Source: J Fungi (Basel). 2024 Nov 21;10(12):807. doi: 10.3390/jof10120807 (PMC11677402; doi:10.3390/jof10120807)
Supplement: Supplementary file 1 [file jof-10-00807-s001.zip › jof-3249447-supplementary.pdf]

Supplementary Material

S1. Effect of temperature on microbial growth of the strain Curvularia kusanoi L7 in different culture media

**Grow of *Curvularia kusanoi* L7 strain at 25ºC**			
**Time (Days)**	**Colony diameter (cm)**		
	**Cpeck**	**AMA**	**APA**
**3**	2.7	2.8	1.03
**6**	5.4	4.2	2.1
**9**	7	6.3	3.2
**Grow of *Curvularia kusanoi* L7 strain at 30ºC**			
**3**	3.5	3	1.4
**6**	6.1	5.4	2.5
**9**	Complete grow	Complete grow	4.5

The values presented in the table correspond to the arithmetic mean of the three determinations made for each schedule by means of culture.

Supplementary Material

S2. Bromatological composition of the fibrous substrates used in the study of enzymatic activity of the strain Curvularia kusanoi L7

Cereal Allbran-Kellogg´s

Composition	Content (%)
Proteins	13
Fats	3
Carbohydrates	40
Sugars	17
Dietary fiber	26.5
sodium	0.5

Fibrous Fractionation of Raw Wheat Straw

Composition	Content (%)
Neutral detergent fiber	67.54
Acid detergent fiber	42.03
Lignin	7.13
Cellulose	28.02
Hemicellulose	25.51

Fibrous fractionation of sugarcane bagasse

Composition	Content (%)
Neutral detergent fiber	49.88
Acid detergent fiber	51.02
Lignin	9.93
Cellulose	36.49
Hemicellulose	12.28

Allbran-Kellogg's cereal is a commercial product and the fibrous substrates sugarcane bagasse and raw wheat straw are agro-industrial waste, previously dried and ground

Structurally, the fiber composition of sugarcane bagasse presents higher values of acid detergent fiber, so its degradation is more complex unlike raw wheat straw which presents a higher value of neutral detergent fiber where hemicellulose is found, which is easier to degrade.

The bromatological analyses of raw wheat straw and sugarcane bagasse were carried out according to what was referred to in the AOAC 1995 and the fiber fractionation according to what was proposed by Goerin and Van Soest, 1970
